# Evaluation of environmental antibiotic contamination by surface wipe sampling in a large care centre

**DOI:** 10.1093/jac/dkae159

**Published:** 2024-06-03

**Authors:** Paul Sessink, Birgit Tans, David Devolder, Rik Schrijvers, Isabel Spriet

**Affiliations:** Exposure Control Sweden AB, Bohus-Björkö, Sweden; Pharmacy Department, University Hospitals Leuven, Leuven, Belgium; Pharmacy Department, University Hospitals Leuven, Leuven, Belgium; Department of Microbiology, Immunology, and Transplantation, Allergy and Clinical Immunology Research Group, KU Leuven, Leuven, Belgium; Department General Internal Medicine-Allergy and Clinical Immunology, University Hospitals Leuven, Leuven, Belgium; Pharmacy Department, University Hospitals Leuven, Leuven, Belgium; Department of Pharmaceutical and Pharmacological Sciences, University Leuven, Leuven, Belgium

## Abstract

**Objectives:**

Exposure of healthcare workers to antibiotics may cause adverse health effects. Results of environmental contamination with antibiotics, obtained by taking surface wipe samples, can be used as an indicator for potential exposure to these sensitizing drugs. The objective was to describe the results of repeated measurements of contamination with antibiotics on multiple surfaces in hospital wards. Standardized needle and syringe preparation techniques and cleaning procedures were used.

**Methods:**

The preparation table and the floor around the waste bin in six wards were sampled and analysed for contamination with the antibiotics amoxicillin, benzylpenicillin, cefotaxime, ceftriaxone, flucloxacillin, meropenem, piperacillin and vancomycin. Sampling was performed in four trials during 8 months. Depending on the outcome of the trials, the cleaning procedure was adapted. Liquid chromatography with tandem mass spectrometry was used for the analysis of the drugs.

**Results:**

During the four trials, contamination with all eight antibiotics was omnipresent on all preparation tables and floors in the six wards. The highest contamination was found for amoxicillin (1291 ng/cm^2^). Changing the cleaning procedure did not reduce the level of contamination.

**Conclusions:**

Surface contamination with the antibiotics was widespread and most probably caused by spillage during the preparation in combination with an ineffective cleaning procedure. Strategies should be developed and implemented by institutions for safe handling of antibiotics to reduce environmental contamination and potential exposure of healthcare workers to these sensitizing drugs.

## Introduction

Antibiotics are widespread used in hospitals and many healthcare workers, especially nurses and pharmacy technicians, are preparing and administering these drugs daily. Although side effects of antibiotics in patients are well described, little is known about the adverse health effects in healthcare workers occupationally exposed to antibiotics.^1^ Reported effects include acute adverse drug reactions such as hypersensitivity, allergic skin reactions, respiratory symptoms and, although very rarely, anaphylactic shock, but antimicrobial resistance associated with long-term exposure has also been observed.^2–6^

Regulatory agencies have outlined the risks of handling sensitizing drugs and the importance to avoid cross-contamination, especially for penicillin and β-lactam antibiotics.^7^ To avoid the risk of cross-contamination, EU and FDA guidance indicate that (β-lactam) antibiotics should be manufactured in dedicated facilities.^8,9^ Reducing environmental contamination from antibiotics (e.g. in air) is also mentioned in the Global Action Plan on Antimicrobial Resistance, published by the World Health Organization in 2015. Environmental contamination is recognized as contributing to the emergence of antimicrobial resistance.^10^ However, no specific protocols exist for handling antibiotics by healthcare workers in hospitals. Mostly, only a hospital protocol for the preparation and administration is available and typically only gloves and/or a face mask are recommended as personal protective equipment (PPE).

Few studies have been published on occupational exposure of healthcare workers to antibiotics. A Swedish study has shown contamination of 12 antibiotics across 21 wards in 16 hospitals.^11^ A recent study in three European hospitals has shown widespread contamination with antibiotics on surfaces and in ambient air where nurses are preparing and administrating antibiotics.^12^ The nurses have also reported smelling the antibiotics, indicating they have been exposed.^12^

The objective of this study was to measure antibiotic surface contamination at different locations in six wards of the University Hospitals Leuven in Belgium. Over an 8 month period, monitoring was repeated four times to get a broader and more longitudinal picture of the contamination than with a single measurement. It was also evaluated whether adjusted cleaning procedures had an impact on the antibiotic contamination.

## Materials and methods

### Study design and site

The study was set up as a prospective observational study, and performed between 26 April and 31 December 2021, in the University Hospitals Leuven, Belgium, a 1950-bed academic tertiary care centre. Over the 8 month period, surface wipe sampling was repeated four times, to get a longitudinal insight in antibiotic surface contamination. Besides, cleaning procedures were adjusted the period before each measurement, to evaluate potential impact on the contamination levels. Ethical approval was not applicable as there was no involvement of patients and nurses.

At the University Hospitals Leuven, antimicrobial stewardship is carried out based on a multifaceted approach. Antimicrobial guidelines including information on reconstitution, dilution and administration of antibiotics, are updated regularly and can be accessed via a locally hosted platform.^13^

### Selection of antibiotics and wards

Contamination with the antibiotics amoxicillin(-clavulanic acid), benzylpenicillin, cefotaxime, ceftriaxone, flucloxacillin, meropenem, piperacillin(-tazobactam), and vancomycin was measured by surface wipe sampling at six hospital wards.

The wards evaluated were a paediatric oncology/haematology ward (20 beds), an adult haematology ward (15 beds), pulmonology (28 beds), a surgical intensive care unit (17 beds), general internal medicine (28 beds) and orthopaedics (28 beds). These wards were selected as they represent wards with a high frequency of patients suffering from infections, e.g. neutropenic fever, bacteraemia, pneumonia, urinary tract infections, osteomyelitis, fracture-related infections and prosthesis-related infections. Consequently, antibiotic treatment is carried out very frequently at these wards.

An overview of the total number of antibiotic vials processed by the nurses per ward on an annual basis is presented in Table 1. When standard dosing in (adult) patients with a normal kidney function is applied, nurses handle one vial (for ceftriaxone 2 g), two vials (for vancomycin 1 g), three vials (for meropenem 1 g and for cefotaxime 2 g) or at least four vials (for piperacillin-tazobactam 4 g, for amoxicillin 1 g, for flucloxacillin 1 g and for benzylpenicillin 1–2 MIU) daily.

**Table 1.** Mean annual numbers of antibiotic vials used in the wards (2019–2021)

Ward	Vancomycin	Cefotaxime	Ceftriaxone	Benzylpenicillin	Amoxicillin	Meropenem	Piperacillin	Flucloxacillin	All eight antibiotics
A: Paediatric oncology/haematology	4256	330	51	27	456	3184	354	158	8816
B: Adult haematology	3588	64	125	9	1560	2596	1728	488	10 158
C: Pulmonology	4672	49	96	0	1869	4395	4270	574	15 925
D: Intensive care unit	5330	538	136	105	2512	3612	4889	846	17 968
E: General internal medicine	1741	293	435	866	2245	1110	2493	2156	11 339
F: Orthopaedics	690	16	18	13	487	28	638	314	2204
All six wards	20 277	1290	861	1020	9129	14 925	14 372	4536	66 410

The antibiotics are prepared by the nurses using needle and syringe technique on the tables being sampled. All antibiotics monitored are dispensed as lyophilized powder in vials. First, the powder is reconstituted with 10 or 20 mL of water for injection or normal saline. Second, depending on the specific antibiotic, the concentrated antibiotic solution is further diluted with normal saline to 50 mL (for syringes at the intensive care unit) or 100–250 mL infusion bags (for the other wards), followed by administration to the patient. For this study, the nurses wore standard (no additional) working clothes and used only gloves as PPE.

### Sampling surfaces, sampling schedule and cleaning procedures

Two identical surfaces were monitored at each ward, i.e. the preparation table (High Pressure Laminate) and floor (Linoleum) around the waste bin in which empty vials were discarded (Table 2). The wipe samples were obtained using AB Wipe Kits from Exposure Control Sweden AB.^14^ The kits contained materials (paper tissues, droppers with distilled water, containers for sample storage and gloves for personal protection) to take wipe samples from several types of surface.

**Table 2.** Surface contamination with eight antibiotics in six wards during four trials (ng/cm^2^)

Ward	Description surface	Surface (cm^2^)	Vancomycin				Cefotaxime				Ceftriaxone				Benzylpenicillin				Amoxicillin				Meropenem				Piperacillin				Flucloxacillin			
			1	2	3	4	1	2	3	4	1	2	3	4	1	2	3	4	1	2	3	4	1	2	3	4	1	2	3	4	1	2	3	4
A: Paediatric oncology/haematology	Preparation table	900	2.45	1.55	155	1.14	ND	ND	ND	973	ND	ND	ND	ND	ND	ND	ND	ND	ND	ND	0.18	ND	19	293	0.31	0.09	0.03	ND	44	ND	ND	ND	ND	ND
	Floor around waste bin	900	4.36	1.28	299	96	0.07	0.10	0.14	49	ND	ND	ND	ND	ND	ND	ND	ND	ND	ND	20	0.33	1.83	2.32	1.48	3.06	0.15	0.20	1.61	0.05	0.01	0.01	2.53	0.15
B: Adult haematology	Preparation table	1054	1.13	0.14	14	4.56	ND	ND	ND	0.04	0.04	ND	ND	ND	ND	ND	ND	ND	14	6.24	ND	0.74	0.07	0.03	0.74	0.07	12	12	0.31	10	ND	ND	ND	16
	Floor Around waste bin	900	1.79	0.70	16	3.16	ND	ND	ND	0.06	0.04	ND	0.11	ND	ND	ND	ND	ND	122	3.40	ND	44	74	1.64	3.13	44	159	47	1.27	44	0.62	0.12	ND	194
C: Pulmonology	Preparation table	3500	2.63	2.63	1.17	0.24	ND	ND	ND	0.01	0.09	0.11	ND	ND	ND	ND	ND	ND	5.43	1.52	0.07	0.06	0.39	0.21	0.03	0.01	1.08	0.85	0.42	0.55	0.01	0.01	0.01	0.02
	Floor around waste bin	900	0.26	0.21	0.19	0.30	ND	ND	ND	0.03	0.04	ND	ND	ND	ND	ND	ND	ND	347	101	0.43	0.93	7.36	3.70	ND	0.04	26	18	0.81	2.92	0.19	0.09	0.03	0.02
D: Intensive care unit	Preparation table	500	0.53	0.26	16	68	ND	ND	1.25	ND	ND	ND	ND	ND	ND	ND	ND	ND	0.05	ND	0.33	0.03	0.38	0.15	0.20	0.66	0.12	0.11	0.40	0.12	0.03	ND	ND	12
	Floor around waste bin	500	4.63	1.52	7.26	1.25	0.03	ND	0.41	ND	0.11	0.06	0.25	ND	ND	ND	ND	ND	1.04	0.38	0.81	ND	2.70	0.80	0.23	1.27	1.75	1.47	0.57	0.24	ND	ND	ND	0.69
E: General internal medicine	Preparation table	500	0.74	3.41	2.97	1.53	0.51	ND	0.65	53	0.11	ND	1.51	ND	ND	5.73	1.94	665	9.16	2.61	9.76	40	0.09	0.03	0.74	0.04	26	22	21	8.15	9.60	0.13	11	1.21
	Floor around waste bin	500	ND	1.49	12	4.68	ND	ND	ND	22	ND	ND	0.09	53	ND	2.80	0.13	119	ND	0.75	7.59	1291	ND	ND	0.56	0.90	0.07	26	0.89	1178	0.30	1.13	3.28	0.25
F: Orthopaedics	Preparation table	500	8.12	0.24	45	22	ND	0.21	ND	ND	ND	0.11	ND	8.98	63	ND	ND	0.50	17	2.44	4.34	0.30	0.04	0.04	0.52	2.66	16	15	0.53	8.73	0.37	7.95	30	0.31
	Floor around waste bin	500	14	0.14	14	191	ND	ND	ND	ND	0.05	ND	ND	0.37	59	ND	ND	21	7.23	0.09	1.14	0.36	ND	ND	0.22	0.53	140	57	4.86	158	5.20	1.05	115	0.56

ND, not detected.

**Figure 1.**
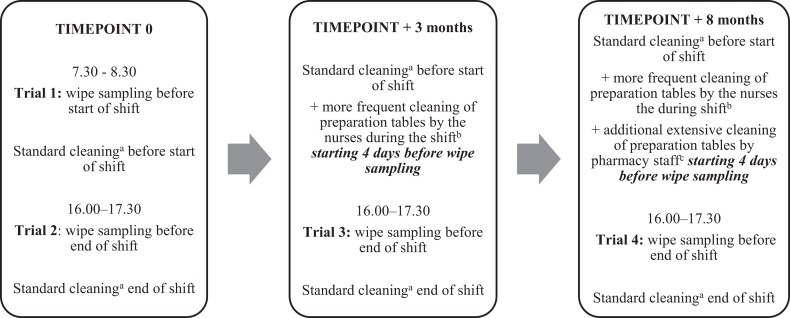
Overview of the study set-up presenting the trials 1–4 at three timepoints, including adjustments in cleaning procedures. ^a^Standard cleaning was carried out with 1% Incidin^®^ Plus (tables) and PolGreen Odor Line Indoors (floors). ^b^Cleaning with Dreft Original. ^c^Cleaning with Dreft Original followed by 70% alcohol.

As shown in Figure 1, wipe samples were taken between 7.30 and 8.30 in the morning before start of the shift (trial 1), and in the afternoon between 16.00 and 17.30 at the end of the shift before cleaning (trial 2). Wipe sampling before cleaning at the end of the shift was repeated 3 months later (trial 3) and 8 months later (trial 4). Per standard protocol, the tables were cleaned with 1% Incidin^®^ Plus while PolGreen Odor Line Indoors was used to clean the floors.^15,16^ After trial 2, preparation protocols and procedures were not changed but cleaning was performed more frequently during the shift using an alkaline diluted household cleaning product (Dreft Original, Procter & Gamble, Belgium), combining alkaline and surface-active characteristics. After trial 3, additional extensive cleaning under supervision of pharmacy staff was performed using the same alkaline detergent, followed by 70% alcohol. The additional cleaning was only performed on the preparation tables, not on the floors.

### Surface wipe sampling, storage, transport and sample preparation

The wipe samples were taken by or under the supervision of an experienced hospital pharmacist. The dimensions of the surfaces were measured and the areas calculated. All samples were stored at −20°C after sampling, during transport and at the laboratory until sample preparation and analysis. The wipe samples were prepared for analysis by adding distilled water. Total extraction volume was 100 mL. After extraction, a part of the extract was used for analysis. The antibiotics were analysed with liquid chromatography and tandem mass spectrometry, as described next. Finally, the contamination in ng per cm^2^ was calculated, a common expression in surface wipe sampling to compare the level of contamination between the surfaces within the study and between published studies. In addition, the contamination score was calculated. The score was 100% if all eight antibiotics were detected on all 12 surfaces during the four trials (8 drugs multiplied with 12 surfaces multiplied by 4 trials gains 384 outcomes).

### Liquid chromatography with tandem mass spectrometry analysis

Analysis was performed on a Xevo TQ-S micro mass spectrometer combined with an Acquity UPLC H-class sample manager and quaternary solvent manager controlled by Masslynx software (Waters, Milford, USA). An Acquity BEH C18, 1.7 µm, 2.1 × 100 mm separation column (Waters, Milford, USA) operated at 40°C was used.

Elution (0.35 mL/min) started with a composition of 100% solvent A (100% MilliQ RO-water with 0.1% formic acid) and 0% solvent B (100% acetonitrile with 0.1% formic acid) with a delay of 2 minutes. Between 2 and 7 minutes, the composition changed to 100% B and from 7.5 minutes starting conditions were restored. Total runtime was 11 minutes, and the injection volume was 8 µL. The mass spectrometer was operated with a capillary voltage of +3 KV, a desolvation temperature of 500°C and a nitrogen flow of 1100 L/hour. Cone gas flow was set at 50 L/min (nitrogen). Argon was used as collision gas. All transitions were measured in positive mode. The antibiotics had a linear calibration curve up to 100 ng/mL. The detection limit was 0.1 ng/mL for amoxicillin and flucloxacillin, and 0.15 ng/mL for the other antibiotics.

## Results

The overall results of the four trials show total spread of contamination with antibiotics up until 1291 ng/cm^2^ (Table 2). Two hundred and fifty-three out of 384 possible outcomes were positive indicating a contamination score of 66%. Contamination scores per ward for each of the trials are presented in Table 3 indicating some differences between the wards and between the trials.

**Table 3.** Contamination score per ward for the four trials (%)

Ward	Trial 1	Trial 2	Trial 3	Trial 4	All four trials
A: Paediatric oncology/haematology	50	44	63	56	53
B: Adult haematology	69	56	44	75	61
C: Pulmonology	75	69	56	75	69
D: Intensive care unit	69	50	69	56	61
E: General internal medicine	56	69	94	94	78
F: Orthopaedics	75	69	63	88	73
All six wards	66	59	65	74	66

For both trials 1 and 2, all surfaces were contaminated with two to seven antibiotics indicating total spread of contamination of the antibiotics at the six wards. Piperacillin and vancomycin were found on all surfaces followed by meropenem, amoxicillin and flucloxacillin. Cefotaxime, ceftriaxone and benzylpenicillin were observed less frequently, and mostly in a few wards. The highest contamination was observed for piperacillin, amoxicillin and meropenem, followed by benzylpenicillin, vancomycin and flucloxacillin. The lowest contamination was found for cefotaxime and ceftriaxone. Considering the overall contamination totalling the concentrations of all eight antibiotics on preparation tables and floors, the median contamination can be ranked as follows: ward E > ward F > ward C > ward B > ward D > ward A. In general, the contamination is comparable in both trials.

For trial 3, all surfaces were contaminated with three to eight antibiotics indicating total spread of contamination on the six wards. Piperacillin and vancomycin were found on all surfaces followed by meropenem, amoxicillin and flucloxacillin. Cefotaxime, ceftriaxone and benzylpenicillin were observed less frequently, and mostly in a few wards. The highest contamination was observed for vancomycin, flucloxacillin, piperacillin and amoxicillin followed by meropenem. The lowest contamination was found for cefotaxime, ceftriaxone and benzylpenicillin. Considering the total contamination of the eight antibiotics on preparation tables and floors, the median contamination can be ranked as follows: ward E > ward F > ward D > ward A > ward C > ward B. In general, the contamination has increased for vancomycin, amoxicillin, meropenem and flucloxacillin in trial 3 compared to trial 2. The contamination has decreased for piperacillin, and was unchanged for cefotaxime, ceftriaxone and benzylpenicillin. Increase of contamination in trial 3 compared to trial 2 was observed for wards A, D, E and F. A decrease was observed in ward C, while no changes were found in ward B.

For trial 4, all surfaces were contaminated with three to eight antibiotics indicating total spread of contamination on the six wards. Meropenem and vancomycin were found on all surfaces followed by piperacillin, flucloxacillin, amoxicillin and cefotaxime. Benzylpenicillin and ceftriaxone were observed less frequently, and only on wards E and F. The highest contamination was observed for vancomycin, and cefotaxime on four surfaces, for benzylpenicillin, flucloxacillin, piperacillin and amoxicillin on three surfaces, and for ceftriaxone and meropenem on one surface. Considering the total contamination of the eight antibiotics on preparation tables and floors, the median contamination can be ranked as follows: ward E > ward B > ward F > ward A > ward D > ward C. In general, the contamination increased for cefotaxime, meropenem, piperacillin and flucloxacillin in trial 4 compared to trial 3. The contamination decreased for vancomycin, and amoxicillin, and was unchanged for ceftriaxone, and benzylpenicillin. However, it should be noted that this was not the case for vancomycin at ward F, for benzylpenicillin, ceftriaxone and amoxicillin at ward E and for amoxicillin at ward B. These exceptions show a substantial increase of the contamination compared to trial 3. In general, an increase of the contamination in trial 4 compared to trial 3 was observed for the wards B, and E. A decrease was observed for the wards A, and D whereas no changes were found for the wards C, and F.

### Discussion and conclusions

In this study, evaluating environmental contamination with antibiotics on a large scale in a university hospital setting, it is shown that contamination with up to eight antibiotics is widespread with high levels of contamination on the preparation tables and on the floors, located next to waste bins. Cleaning appeared to be ineffective as already in trial 1, before start of the shift, substantial and widespread contamination was found. Contamination continues, also in trial 3, when more frequent cleaning with an alkaline and surface-active detergent was performed during the shift. Therefore, additional extensive cleaning was performed under the supervision of the pharmacy staff before trial 4 was conducted. However, the contamination was not reduced and seems to be persistent and difficult to remove once present.

It is obvious that the contamination is caused by the preparation technique. The use of the needle and syringe technique for reconstitution of the lyophilized powder and subsequent dilution in the infusion fluid has resulted in spillage due to overpressure in the drug vials and during withdrawal of the needle-syringe from the vials. In addition, spiking of infusion bags might also cause spillage. Consequently, the preparation area (table and floor around the waste bin) gets contaminated. However, additional contamination from (the exterior of the) drug vials cannot be excluded.^17,18^ Vials were not checked for contamination on the outside and it is possible that contamination on the vials has been transferred e.g. by hands or gloves to other surfaces including the preparation tables.

As far as we know, two studies have been published measuring surface contamination with antibiotics. A Swedish study has shown contamination of 12 antibiotics across 21 wards in 16 hospitals.^11^ Half of the wards had contamination levels >0.5 ng/cm^2^. The lowest contamination was observed for the wards using a closed-system drug-transfer device (CSTD). In the other study three European hospitals were involved.^12^ Widespread contamination with seven antibiotics up to 767 ng/cm^2^ was found in the wards. After CSTD implementation, contamination levels were significantly decreased.

According to the National Institute for Occupational Safety and Health, a CSTD is defined as a drug-transfer device that mechanically prohibits the transfer of environmental contaminants into the system and the escape of the hazardous drug or vapour concentrations outside the system.^19^ USP <800> recommends the use of CSTDs for hazardous drug compounding but CSTDs are required during hazardous drug administration.^20^ As CSTDs offer enhanced protection to healthcare workers, they are routinely used, also in our centre, for compounding and administration of hazardous drugs, in combination with other protective measures such as biohazard cabinets and PPE.^21–27^ It is expected that the contamination with antibiotics found in the present study will also be reduced after implementation of a CSTD. It is obvious that hospital wide implementation of a CSTD for antibiotics will have a major financial impact as antibiotics are estimated to be used in one-third of the hospitalized patients.^28^

The surface wipe sample results clearly show high levels of environmental contamination with antibiotics and, hence, it is expected that contaminated surfaces might result in exposure of the healthcare workers. An overview of studies published between 2000 and 2023 evaluating whether exposure might lead to adverse health effects in healthcare workers is presented in Tables 4 and 5. Both immediate and delayed-type hypersensitivity reactions after occupational exposure have been described. In case of type I (IgE-mediated) sensitization, contact urticaria up to anaphylaxis, even after airborne exposure, have been described. In case of a type IV (T-cell mediated) sensitization pattern, the clinical presentation was mostly occupational allergic contact dermatitis (OACD) affecting the hands and to a lesser extent forearms or face, the last indicating airborne dermatitis. Sensitization was not always accompanied by symptoms, and it remains unknown to what extent systemic exposure would induce reactions in the evaluated healthcare workers.^2,3,37^ The prevalence of penicillin sensitization in these studies decreased, paralleling a decrease in use.^2,31^ The sensitization rate to the evaluated antibiotics in our study remains unknown. Recent work suggested a sensitization rate of only 1% for antibiotic-induced OACD among referred healthcare workers,^29^ although studies systematically evaluating all healthcare workers reported sensitization rates from 0.7 up to 12% (2.6%–12% for type I and 0.7%–9.8% for type IV).^2,3,31,37^ It is less clear, and difficult to investigate, what the impact is of long-term exposure to broad-spectrum antimicrobials on e.g. the development of antimicrobial resistance of the skin microbiome. Ideally, both types of exposure should be avoided by healthcare workers.

**Table 4.** OACD to antibiotics reported in literature

Author (year), country	Population (*n*)	Testing procedure	Results	Remarks
Gilissen *et al*. (2019), Belgium^29^	HCWs (*n* = 201) 201 OACD patients out of 1248 HCWs examined 2001–2019.	Patch	26/201 (13%) had OACD to systemic drug(s), 3 (1%) for antibiotics (2 BL).	Hands were mostly affected, but also face (airborne dermatitis) was observed.
Pinheiro *et al*. (2018), Portugal^4^	HCWs (*n* = 4) Monocentric, retrospective description of OACD to systemic antibiotics in HCWs, 2010–2016.	Patch	4 cases with OACD to cephalosporins (3) and/or penicillins (2). One patient with pre-existing DRESS after (systemic exposure to) flucloxacillin.	Hands (mainly dorsa), forearms and face were affected. Generalized rash on airborne exposure was observed.
Yesudian and King (2001), UK^30^	HCW (*n* = 1) Case report.	Patch	OACD (face) due to meropenem.	
Rudzki *et al*. (1999), Poland^31^	HCW (not reported) Monocentric, retrospective data of percentage positive penicillin patch tests among nurses, 1971–1998.	Patch	Sensitization rate among nurses with OACD was 0.7% to 9.8%.	A decreasing trend in sensitization rate from 1990 onwards was observed.
Foti *et al*. (1997), Italy^32^	HCW (*n* = 18) Monocentric case series with OACD with exposure to cephalosporins (nurses).	Patch, SPT, IDT	8/18 (44%) showed positive patch testing.	Hands (18 cases), forearms (3 cases), face (2 cases) were affected.

HCW, healthcare worker; BL, ß-lactams; DRESS, drug reaction with eosinophilia and systemic symptoms; SPT, skin prick testing; IDT, intradermal skin testing.

**Table 5.** Occupational IgE-mediated allergy to systemically used antibiotics

Author (year), country	Population (*n*)	Testing procedure	Results	Remarks
Gaspar-Marques *et al*. (2018), Portugal^33^	HCW (*n* = 1) Case report, nurse.	SPT, IDT	Recurrent anaphylaxis at work and after piperacillin-tazobactam preparation with positive skin tests for penicillins, cefuroxime.	Airborne exposure-induced anaphylaxis.
Merget *et al*. (2017), Germany^34^	HCW (*n* = 1) Case report, nurse.	SPT, IDT, sIgE, BAT, inhalation provocation	Recurrent anaphylaxis; severe anaphylaxis after oral cefuroxime exposure.	Inhalation of 10 μg cefuroxime induced generalized urticaria.
Classen and Fuchs (2015), Germany^35^	HCW (*n* = 1) Case report, nurse.	SPT, IDT, open Patch, rub test	Recurrent contact urticaria when preparing BL antibiotics.	
Kim *et al*. (2012), South Korea^3^	HCW (*n* = 161) Recruitment from a single tertiary centre.	SPT to cefotiam, ceftriaxone, ceftizoxime	Sensitization rate was 3.1%.	Also sIgE determination was performed showing a 17.4% sensitization prevalence (but relevance is debatable). Also 86 non-atopic non-allergic controls included but without data on SPT results.
Kim *et al*. (2011), South Korea^36^	HWC (*n* = 1) Case report, nurse.	sIgE testing	Anaphylaxis after administration of piperacillin-tazobactam.	Pre-existing atopic dermatitis.
Choi *et al*. (2010), South Korea^37^	HCW (*n* = 311) 457 out of 539 staff nurses at a tertiary centre responded to a questionnaire, 318 received skin testing, 311 were analysed.	SPT (cefotiam, cefoperazone, ceftizoxime, flomoxef, piperacillin, penicillin G	Sensitization rate was 2.6% (8 out of 311). Symptoms of contact urticaria were present in 5/8 HCWs. In 7/8 for at least one penicillin, 6/8 for at least one cephalosporin.	Sensitization was more prevalent in those with versus without a pre-existing skin disorders (atopic dermatitis, urticaria, contact dermatitis) (6.9% versus 1.3%, *P* = 0.018).
Cetinkaya *et al*. (2007), Turkey^2^	HCWs (*n* = 83) Nurses handling beta-lactam antibiotics.	SPT, IDT (PPL, MDM)	10 (12%) had positive skin tests.	None had reacted but limited/no exposure. Interpreted as occult sensitization.

HCW, healthcare worker; SPT, skin prick testing; IDT, intradermal skin testing; sIgE, specific IgE; BAT, basophil activation test; PPL, penicilloyl polylysine; MDM, minor determinant mixture.

Currently there are no recommendations for safe handling, cleaning or risk assessment for surface contamination with antibiotics. Determination of contamination at individual facilities via regular monitoring programmes as performed in this study may identify potential exposure of healthcare workers and patients to these harmful drugs. Update of cleaning procedures, including alternative or additional cleaning agents, may reduce the antibiotic surface contamination but requires assessment of its effectiveness as performed for hazardous drugs.^38^ Ultimately, implementation of engineering controls such as CSTDs or similar devices may provide the most effective method to reduce or eliminate the environmental contamination and the exposure risk. As no specific official regulations for antibiotics exist, it is recommended that legal authorities and professional organizations of hospital pharmacists and nurses take the initiative to develop these to ensure safety of the healthcare workers. The European Centre for Disease Prevention and Control and the European Society of Clinical Microbiology and Infectious Diseases could be other relevant stakeholders in this process.

The main limitation of our study concerns the uncertainty to determine the actual levels of contamination for the antibiotics on the different surfaces. The main issue is the wipe efficiency depending on type of surface, liquid, paper tissue and the chemical and physical characteristics of the antibiotics. A single wipe test as performed will not be able to remove all residual surface contamination especially when it concerns rough and sticky surfaces. Only smooth surfaces such as plastics and stainless steel will give high recoveries and reliable results. Recovery and wipe efficiency have been validated for seven antibiotics on plastic and stainless steel and ranged from 21% for piperacillin to 94% for cefotaxime.^12^ The solubility of the antibiotics in water is high and consequently the extraction from the tissue is very efficient. As there are many variables involved, it is impossible to determine the exact recovery for each antibiotic on all types of surface. Hence, the results presented are not corrected for recovery. Consequently, the actual surface contamination will be higher than the measured contamination.

Other limitations are the absence of statistical analysis, assessment of stability of antibiotics during storage of wipes and lack of reference values for surface contamination. Statistical analysis is lacking as we decided to perform a descriptive study based on four trials. The focus was to explore antibiotic contamination hospital wide and not to look for differences between wards, antibiotics and surfaces monitored. Potential degradation of the antibiotics, e.g. meropenem, during storage of the wipes in the freezer was not monitored.^39^ Currently, there are no upper limit thresholds for antibiotic surface contamination based on a risk assessment related to adverse health effects. Lacking reference values makes interpretation of the contamination levels difficult. When compared to previous measurements for hazardous drugs, antibiotic contamination levels are markedly higher, probably related to the fact that antibiotic doses are typically from 100- up to 1000-fold higher compared to hazardous drugs.^27^ As antibiotics are considered sensitizing drugs, the precautionary principle should be applied, i.e. exposure should be minimized as much as possible.

In conclusion, surface contamination with antibiotics is probably caused by spillage during preparation in combination with an ineffective cleaning procedure. The widespread contamination requires attention to avoid potential adverse health effects for healthcare workers. Future research should focus on the impact of CSTD implementation and effective cleaning procedures.
